# The effect of acupoint stimulation as an adjuvant therapy for obsessive-compulsive disorder: a systematic review and meta-analysis

**DOI:** 10.3389/fneur.2026.1875285

**Published:** 2026-07-23

**Authors:** Jiangwei Yang, Zhen Liu, Zixin Liang, Xiaochun Jiang, Xinxin Jiang, Honglian Xia

**Affiliations:** 1 Tongde Hospital of Zhejiang Province, Mental Health Center of Zhejiang Province, Hangzhou, Zhejiang, China; 2 The Third Clinical Medical College, Zhejiang Chinese Medical University, Hangzhou, Zhejiang, China; 3 The Third School of Clinical Medicine (School of Rehabilitation Medicine), Zhejiang Chinese Medical University, Hangzhou, Zhejiang, China; 4 The Second Affiliated Hospital of Zhejiang University School of Medicine, Hangzhou, Zhejiang, China; 5 Shaoxing Seventh People's Hospital (Affiliated Mental Health Center, Medical College of Shaoxing University), Shaoxing, Zhejiang, China

**Keywords:** acupoint stimulation, adjuvant therapy, compulsive behavior, meta-analysis, obsessive-compulsive disorder

## Abstract

**Background:**

Obsessive-compulsive disorder (OCD) is a debilitating psychiatric condition associated with significant functional impairment. Acupoint stimulation (AS), encompassing techniques such as electroacupuncture, acupuncture, and transcutaneous electrical acupoint stimulation, is a widely used therapeutic approach. However, a comprehensive synthesis of evidence regarding its efficacy and safety as an adjunctive therapy for OCD is needed.

**Objective:**

This systematic review and meta-analysis aimed to evaluate the efficacy and safety of AS as an adjunctive treatment for OCD.

**Methods:**

A comprehensive literature search was conducted across seven electronic databases from their inception to February 2026. Randomized controlled trials (RCTs) investigating AS for OCD were included. Two reviewers independently screened records, extracted data, and assessed methodological quality using the Cochrane Risk of Bias tool (ROB 2.0). Statistical analyses were performed using Review Manager 5.4.

**Results:**

This review included 23 RCTs involving 1,713 participants. Compared with the controls, AS was associated with greater improvement in OCD. It was associated with a statistically significant reduction in obsessive-compulsive symptoms as measured by the Yale-Brown Obsessive Compulsive Scale (Y-BOCS) (MD = −3.14, 95% CI [−4.46, −1.81], *p* < 0.00001) and also associated with improvements in anxiety and Clinical Global Impression (CGI) scores, and other secondary outcomes. The combination of AS with both pharmacotherapy and CBT yielded the largest Y-BOCS reduction among subgroups examined (3 studies; MD –3.50, 95% CI [−4.34, −2.66]; *p* < 0.00001). Regarding safety, the AS group had fewer reported adverse events than medication controls (RR = 0.17, 95% CI [0.06, 0.51], *p* < 0.0001). Subgroup analyses suggested that acupoint prescriptions containing PC6 and a treatment frequency of 20–40 times may be associated with the largest point estimate, though this comparison is exploratory.

**Conclusion:**

This meta-analysis found that AS, whether used alone or as an adjunct to pharmacotherapy or CBT, was associated with a mean reduction of approximately 3.14 points on the Y-BOCS. On a 0–40 scale, with substantial heterogeneity across studies, variable control conditions, and inconsistent methodological quality, this reduction is statistically significant but of uncertain clinical relevance. The evidence does not support a firm conclusion that AS is clinically effective, and the safety data are insufficient to establish AS as safe. Further high-quality trials with standardized protocols are needed.

**Systematic review registration:**

CRD420251070503.

## Introduction

Obsessive-compulsive disorder (OCD) is a chronic psychiatric condition frequently associated with substantial functional impairment (1). It is characterized by intrusive thoughts or images known as obsessions, and ritualistic mental or physical behaviors performed to alleviate associated anxiety or distress, termed compulsions (2). An epidemiological study has found that the global lifetime prevalence among adults is approximately 1.3%, with males consistently exhibiting a lower risk than females (3). Left untreated, obsessive-compulsive disorder can impair social functioning and significantly diminish quality of life (4). Prior studies have classified OCD symptoms into four distinct, yet overlapping, dimensions, including: (a) contamination and cleaning; (b) symmetry and ordering; (c) forbidden thoughts; and (d) harm (5). However, this broad dimensional summary limits its direct applicability in clinical practice. The Y-BOCS is widely regarded as the gold standard for assessing OCD severity in clinical populations. It differentiates symptom types across specific domains, including aggressive, contamination, sexual, religious, symmetry/exactness, and somatic obsessions, as well as hoarding-related symptoms (6). Despite being ranked among the 10 most debilitating disorders, OCD is characterized by suboptimal treatment-seeking rates. Studies report a mean duration of untreated illness estimated at 17 years following symptom emergence. Consequently, the timely identification of early OCD symptoms and the prompt implementation of effective intervention are of substantial clinical importance (7, 8). Studies indicate that exposure and response prevention (ERP) demonstrates significant efficacy in treating OCD among children, adolescents, and adults, with a treatment response rate of 65 to 70% and a remission rate of 57% (9). Evidence indicates that cognitive-behavioral therapy (CBT), incorporating ERP, is effective in approximately 50% of clinical cases (10, 11). A substantial proportion of patients discontinue treatment prematurely, often due to perceiving exposure exercises as excessively challenging or the induced anxiety as overly intense (12, 13). Furthermore, an estimated 20% of individuals with OCD do not respond to all available treatment modalities (14). Therefore, it is imperative to refine existing treatment approaches and advance alternative therapies to enhance the quality of life for individuals with OCD.

The AS is a prevalent therapeutic approach within Traditional Chinese Medicine. It encompasses various techniques, including acupressure, acupuncture, acupoint injection, finger pressure, transcutaneous electrical acupoint stimulation (TEAS), and moxibustion. AS has been explored in preliminary clinical research and TCM-based literature for OCD, but the evidence remains limited and has not been systematically evaluated (15–18). Crucially, the specific impact of AS as an adjunctive therapy on the mental state and anxiety symptoms of OCD patients remains underexplored in systematic reviews. Conducting a rigorous and up-to-date systematic review addressing this gap is essential. Therefore, the systematic review and meta-analysis focus on evaluating the effects of AS on obsessive-compulsive symptoms, illness severity, and anxiety in OCD.

## Methods

This meta-analysis was conducted in adherence to the PRISMA guidelines and the Cochrane Handbook for Systematic Reviews of Interventions. The study protocol was prospectively registered on PROSPERO under registration number CRD420251070503.

### Resources and searches for data

Seven electronic databases from inception through February 1, 2026: Sinomed (Chinese Biomedical Literature Database), CNKI (China National Knowledge Infrastructure), Wanfang, Embase, Cochrane Library, Medline, and Web of Science. The following describes the search strategy employed in the Medline: (((Transcutaneous Nerve Stimulation) OR (acupoint stimulation)) OR ((((((“Acupuncture Points”[Mesh]) OR (“Acupuncture”[Mesh] OR “Acupuncture Therapy”[Mesh] OR “Acupuncture, Ear”[Mesh])) OR “Meridians”[Mesh]) OR “Acupressure”[Mesh]) OR “Electroacupuncture”[Mesh]) OR “Transcutaneous Electric Nerve Stimulation”[Mesh])) AND ((((Disorder, Obsessive-Compulsive) OR (Anankastic Personality)) OR (Obsessive-Compulsive Neuroses)) OR (“Obsessive-Compulsive Disorder”[Mesh])). No language restrictions were applied. Records were initially screened based on titles and abstracts. Subsequently, the full texts of potentially eligible studies were retrieved for detailed assessment.

### Eligibility criteria

Inclusion studies met the following criteria:

1. Participants: Patients with a confirmed diagnosis of OCD.
2. Interventions: This study will include any form of acupoint stimulation therapy involving electroacupuncture (EA), acupuncture, acupoint injection, acupoint pressure, Transcutaneous Electrical Acupoint Stimulation (TEAS), or moxibustion, or combinations of acupoint stimulation with other treatments. In add-on trial designs where AS was added to a standard-of-care comparator delivered equally to both arms, the independent contribution of AS could be estimated; such studies were eligible.
3. Comparators: The control group should comprise non-acupoint stimulation treatments, including a sham acupoint stimulation group, a drug intervention group, and a physiotherapy group.
4. Outcomes: Primary outcomes: Yale-Brown Obsessive Compulsive Scale (Y-BOCS); Secondary outcomes: include the anxiety state rating scale, Brief Psychiatric Rating Scale (BPRS), and the CGI; Safety Outcome: Incidence of total adverse events. This includes any adverse events and serious adverse events defined in the original study, such as somnolence, loss of appetite, taste disturbances, dizziness, and constipation.
5. Study design: Only RCTs were eligible.
6. Language: No language restrictions. Literature in languages other than English and Chinese was also included. The protocol specified that records in any other language would be translated by a qualified medical translator or evaluated by a native-speaker collaborator before screening and extraction.

Exclusion criteria are as follows:

1. Studies were excluded if AS was one component within a combination of three or more active experimental interventions, such that the specific contribution of AS could not be disentangled.
2. Unavailable data.
3. Full-text research articles were eligible, while conference proceedings, dissertations, and gray literature were excluded.

### Study screening, data collection, and risk of bias

All records were imported into EndNote X9. After initial automated deduplication, manual verification ensured the complete removal of duplicate literature. Two independent reviewers screened titles and abstracts to identify potentially eligible studies. Full texts of these studies were then assessed against the predefined inclusion criteria. Any disagreements were resolved through consensus with a third reviewer.

Two independent reviewers conducted preliminary data extraction and cross-verification, with discrepancies resolved through arbitration by a third reviewer. The extracted general characteristics included: first author, publication year, country, sample size, participant gender, mean age, illness duration, treatment modality, intervention acupoints, treatment duration, and outcome measures. Data on safety outcomes were also extracted.

Methodological quality and risk of bias were rigorously assessed using the revised Cochrane Risk of RoB 2.0. The validated instrument systematically assesses five key domains: (1) the randomization process; (2) deviations from intended interventions; (3) missing outcome data; (4) outcome measurement; and (5) selective reporting of results. Two independent reviewers performed the assessments in parallel. Any discrepancies in bias ratings were resolved through consensus discussion with a third reviewer.

### Statistical analysis

All statistical analyses were performed using Review Manager 5.4. For safety outcomes, the risk ratio (RR) with a 95% confidence interval (CI) was used as the effect measure. For continuous outcomes, the mean difference (MD) with a 95% CI was applied. Where necessary, confidence intervals or interquartile ranges reported in original studies were converted to standard deviations using methods recommended by the Cochrane Handbook.

Statistical heterogeneity was assessed using the *I*^2^ statistic and Cochran’s *Q* test. *I*^2^ values of 25, 50, and 75% represented low, moderate, and high heterogeneity, respectively. A fixed-effect model was applied when heterogeneity was low (*I*^2^ < 50% and *p* > 0.100); otherwise, a random-effects model was used. Subgroup analyses were conducted to explore sources of heterogeneity based on study characteristics such as treatment duration and modality.

A sensitivity analysis was performed by sequentially excluding the three studies with the highest weight to assess the robustness of the pooled results. Publication bias was evaluated using funnel plots if 10 or more RCTs were included.

## Results

### Studies selection

**Figure 1:**
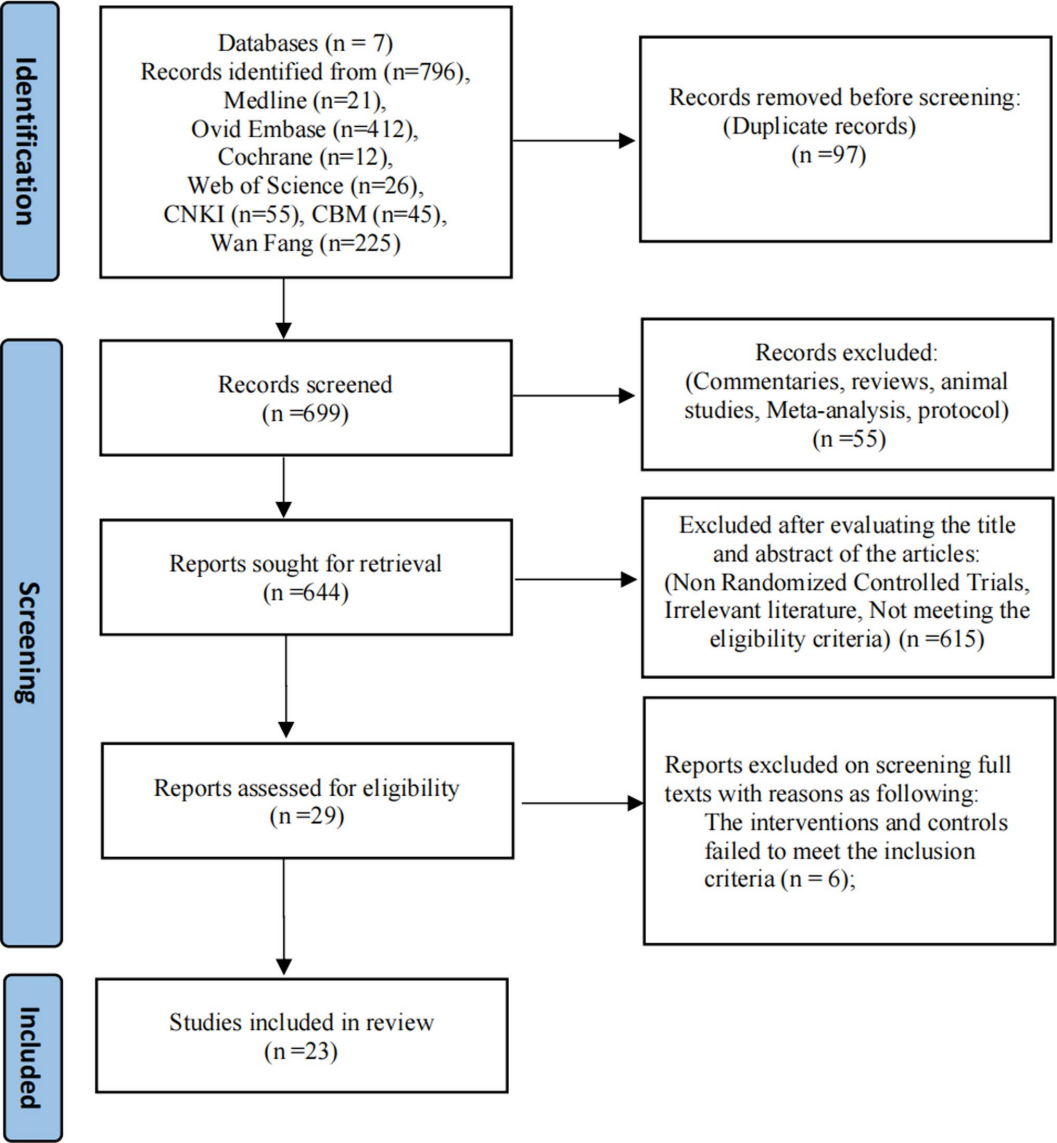
Flow diagram of study screening.

A systematic search of 7 databases yielded 796 records, of which 97 were duplicates. All records retrieved were published in English or Chinese; no records in other languages were identified. Following title and abstract screening, 29 full-text articles were assessed for eligibility. Five of these were excluded due to non-conforming interventions or control conditions. Finally, 23 studies involving 1713 patients were included in the quantitative synthesis, as shown in Figure 1.

### Included studies’ characteristics

Twenty-three RCTs evaluating AS for OCD were included (19–41). All studies were conducted in China, involving 860 patients in treatment groups and 853 in control groups. Thirteen studies had over 40 intervention sessions, with publications spanning 1999 to 2022. The AS modalities employed were acupuncture, electroacupuncture, and TEAS. Study characteristics are summarized in Table 1.

**Table 1:** Characteristics of RCTs.

Author, year, country	Sample size (T/C)	Mean age (years)	Gender (male/female)	Acupoint protocol	Disease duration	Treatment duration	Outcomes
Chen et al. (2008) (22)	EA + Fluoxetine (*n* = 36) Fluoxetine (*n* = 36)	T: 41.37 ± 9.25 C: 38.76 ± 7.85	T: 20/16 C: 18/18	GV20, EX-HN3	T: 15.60 ± 7.78 years C: 12.18 ± 8.74 years	Once daily, for 6 weeks	Y-BOCS
Feng (2001) (23)	EA + Clomipramine (*n* = 15) Clomipramine (*n* = 20)	T: 26 C: 26	T: 9/6 C: 12/8	PC6	T: 5.5 years C: 5.4 years	3 times for 2 weeks	Y-BOCS Safety
Feng et al. (2005) (24)	EA + CBT (*n* = 33) Clomipramine (*n* = 32)	T: 31.79 ± 14.67 C: 29.90 ± 12.80	T:14/18 C:15/18	PC6, PC8, GV20	T: 65 months C: 66 months	Once daily for 8 weeks	Y-BOCS BPRS
Feng et al. (2013) (25)	EA + CBT + Clomipramine (*n* = 81) Sham EA + Clomipramine (*n* = 79)	T: 30.99 ± 12.01 C: 31.41 ± 12.77	T:34/47 C:46/33	PC6	T: 66 months C: 54 months	Once a day, 5 times weekly, for a total of 12 weeks	Y-BOCS BPRS
Feng et al. (2016) (20)	TEAS + CBT + clomipramine (*n* = 120) Sham TEAS + CBT + clomipramine (*n* = 120) Cognitive behavioral therapy (CBT) Transcutaneous electrical acupoint stimulation (TEAS)	T: 33.9 ± 11.7 C: 35.4 ± 12.6	T: 52/68 C: 52/68	PC6	T: 1–10 years C: 1–10 years	Once daily, for 12 weeks	Y-BOCS
Bin et al. (2007) (19)	EA + Clomipramine (*n* = 30) Clomipramine (*n* = 30)	T: 29.90 ± 12.80 C: 28.73 ± 12.87	T: 13/17 C: 13/17	PC6	T: 65 months C: 66 months	Once daily, for 8 weeks	Y-BOCS BPRS Safety
Hu et al. (2009) (26)	Acupuncture (*n* = 34) Clomipramine (*n* = 34)	T: 38.94 ± 13.00 C: 37.82 ± 13.16	T:18/16 C:17/17	PC6, SP6	T: 3.78 ± 5.36 C: 3.56 ± 4.13	Every 2 days for a total of 8 weeks	Y-BOCS Safety
Huang et al. (2005) (27)	EA + Fluoxetine (*n* = 30) Fluoxetine (*n* = 30)	T: 30.4 ± 5.1 C: 32.4 ± 8.9	T: 17/13 C: 19/11	PC8	T: 5.5 ± 2.3 years C: 6.8 ± 5.2 years	Twice a week for 8 weeks	Y-BOCS
Li et al. (2016) (28)	Acupuncture + Paroxetine (*n* = 30) Paroxetine (*n* = 30)	T: 31.93 ± 10.18 C: 32.10 ± 11.11	T: 14/16 C: 15/15	PC6, SP6, GV20, EX-B2	T: 6.3 ± 3.2 years C: 6.7 ± 3.8 years	Once a week for 8 weeks	Y-BOCS
Liang (2016) (29)	Acupuncture + Clomipramine (*n* = 30) Clomipramine (*n* = 30)	T: 45.22 ± 2.94 C: 45.22 ± 2.94	/	GV20, EX-B2, PC6, SP6	T: 10.27 ± 3.24 years C: 10.27 ± 3.24 years	Once a day for 4 weeks	Y-BOCS
Lv (2002) (30)	Acupuncture (*n* = 32) Clomipramine (*n* = 28)	T: 28.3 ± 11.4 C: 26.1 ± 11.6	T: 20/12 C: 16/12	PC6, GV26, SP6	T: 31.02 ± 37.64 months C: 28.31 ± 20.23 months	Once a week for 8 weeks	Y-BOCS Safety
Shu et al. (1999) (31)	EA + Chlorpromazine (*n* = 30) Chlorpromazine (*n* = 30)	T: 25.5 ± 6.2 C: 25.5 ± 6.2	/	EX-HN3, GV20, EX-HN5	T: 3.4 ± 0.75 years C: 3.4 ± 0.75 years	Once a day for 8 weeks	Y-BOCS
Song et al. (2022) (32)	Acupuncture (*n* = 35) paroxetine (*n* = 35)	T: 21 ± 16 C: 22 ± 15	T: 17/18 C: 16/19	GV20, EX-HN3, PC6, HT7, KI6, LR3	T: 18.6 ± 9.3 months C: 19.7 ± 8.9 months	Once a day, 6 times weekly, for a total of 8 weeks	Y-BOCS HAMA
Sun et al. (2004) (33)	EA + fluoxetine (*n* = 15) Fluoxetine (*n* = 15)	T: 33.8 ± 10.44 C: 34.3 ± 12.20	T: 8/7 C: 7/8	PC6, GV26, CV24	T: 3.66 ± 1.85 years C: 3.96 ± 2.10 years	The first 2 weeks: six times weekly; The following 6 weeks: every other day	Y-BOCS
Wang et al. (2009) (34)	EA + Fluvoxoxamine (*n* = 26) Fluvoxoxamine (*n* = 26)	T: 35 ± 5 C: 39 ± 7	T: 15/11 C: 14/12	PC6	T: 19 ± 16 months C: 22 ± 19 months	Once a day for 12 weeks	Y-BOCS CGI Safety
Wu and Ding (2017) (35)	EA + Clomipramine (*n* = 50) Clomipramine (*n* = 50)	T: 30.32 ± 3.30 C: 30.05 ± 3.83	T: 22/28 C: 23/27	PC6, PC8, GV20	T: 1.32 ± 0.41 years C: 1.22 ± 0.38 years	Once a day, 5 times weekly, for a total of 8 weeks	Y-BOCS BPRS Safety
Xiao et al. (2016) (36)	Acupuncture + Paroxetine (*n* = 30) Sham acupuncture + Paroxetine (*n* = 30)	T: 46.64 ± 2.32 C: 5.98 ± 2.09	T: 19/11 C: 19/11	GV20, PC6, EX-HN3, GV26	/	Once a day, 5 times weekly, for a total of 8 weeks	Y-BOCS CGI HAMA
Zhang (2020) (37)	Acupuncture + fluvoxamine (*n* = 25) Fluvoxamine (*n* = 25)	T: 38.88 ± 10.49 C: 38.96 ± 10.52	T: 16/9 C: 15/10	PC6, PC8, LI4, HT7, SP6	T: 3.36 ± 1.78 years C: 3.20 ± 1.82 years	Once a day for 8 weeks	Y-BOCS SAS Safety
Zhang and Liu (2008) (38)	EA + CBT + SSRIs (*n* = 32) SSRIs (*n* = 28)	T: 30.5 ± 7.4 C: 31.2 ± 9.4	T: 15/17 C: 15/13	GV20, EX-HN3	T: 3 months ~ 2 years C: 4 months ~ 2.5 years	3 times weekly for a total of 20 weeks	Y-BOCS
Zhang et al. (2002) (39)	EA + Clomipramine (*n* = 42) Clomipramine (*n* = 42)	T: 38.76 ± 12.67 C: 37.56 ± 11.71	T: 25/17 C: 28/14	GV20, EX-HN3, GV21, GV19, GV17, GV15, EX-HN5	T: 3.72 ± 5.76 years C: 3.66 ± 4.83 years	6 times weekly for a total of 8 weeks	Y-BOCS Safety
Zhang et al. (2015) (40)	Acupuncture + fluvoxamine (*n* = 34) Fluvoxamine (*n* = 34)	T: 34. 8 ± 11.6 C: 36. 3 ± 9.7	T: 16/18 C: 15/19	LI4, PC6, HT7, PC8, SP6	T: 3. 37 ± 1. 63 years C: 2. 91 ± 1. 86 years	Once a day for 8 weeks	Y-BOCS SAS Safety
Zhang and Guo (2017) (41)	Acupuncture + buspirone (*n* = 60) Buspirone (*n* = 60)	T: 29.31 ± 12.58 C: 28.56 ± 11.35	T: 36/24 C: 35/25	GV26, PC6, SP6, EX-B2	T: 2.35 ± 1.12 years C: 2.62 ± 1.76 years	Once a day for 2 months	Y-BOCS HAMA Safety
Zhang et al. (2009) (21)	EA (*n* = 10) Waitlist (*n* = 9)	T: 28.5 ± 12.9 C: 29.9 ± 11.7	T: 4/6 C: 4/5	GV20, EX-HN3, EX-HN1, GB15, GB8, EX-HN5, ST8	T: 9.2 ± 9.9 years C: 7.6 ± 9.0 years	5 times per week for 12 times	Y-BOCS CGI

T, treatment group; C, control group; Y-BOCS, Yale-Brown Obsession Scale; BPRS, Brief Psychiatric Rating Scale; HAMA, Hamilton Anxiety Scale; SAS, Self-Rating Anxiety Scale; CGI, Clinical Global Impression; SSRIs, Selective Serotonin Reuptake Inhibitors.

### Analysis of acupoint combination

**Figure 2:**
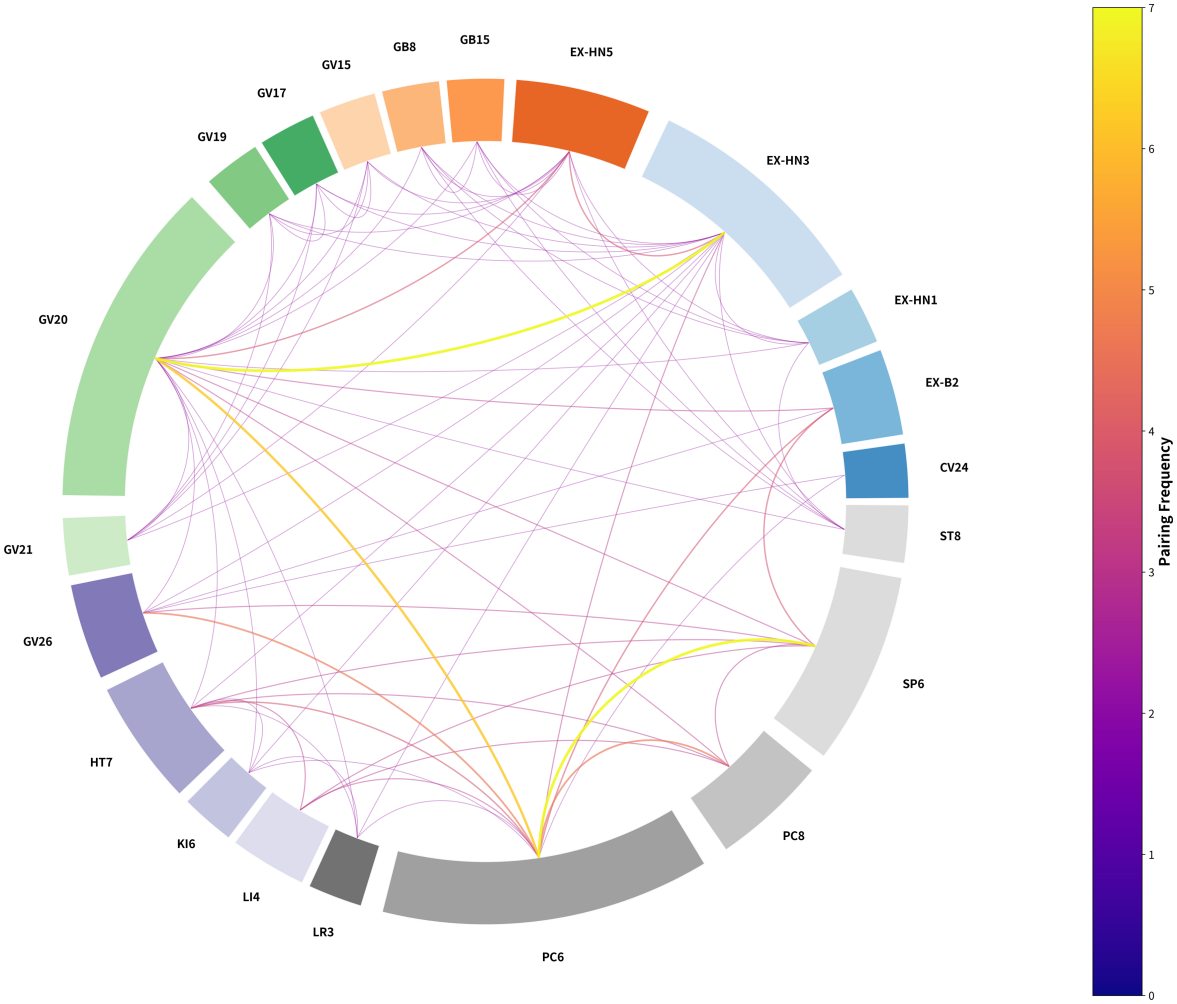
Acupoint combination string diagram.

**Figure 3:**
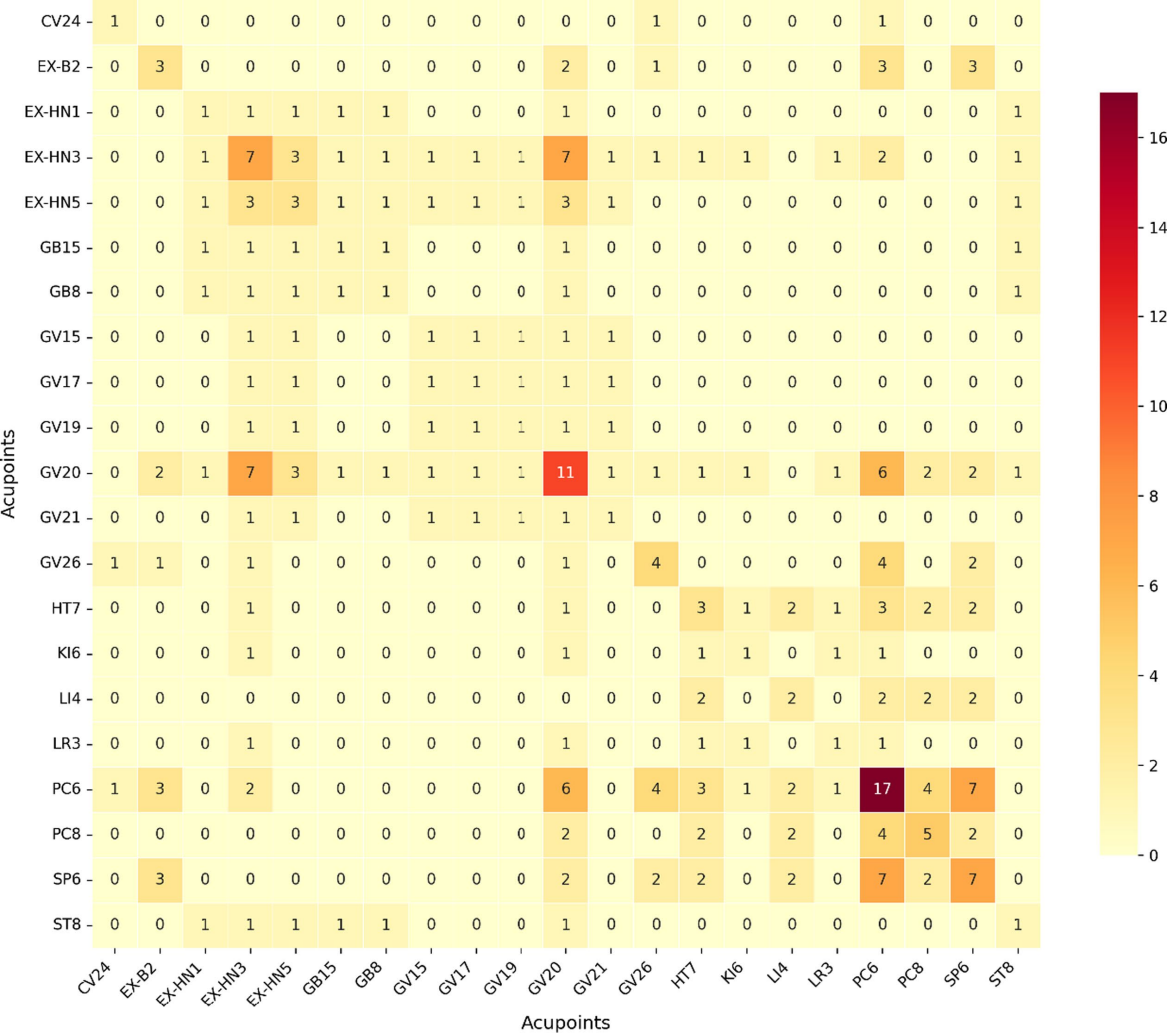
Acupoint combination heat map. For the heat map, diagonal values represent the total frequency with which a given acupoint appeared across all 23 included protocols. Off-diagonal values represent the number of protocols in which both acupoints co-occurred.

We also analyzed patterns of acupoint combinations and identified the acupoints that were used most frequently. PC6 was found to be the most commonly needled point, featuring in 17 combinations. GV20 followed closely behind, featuring in 11 combinations. Other points used more than seven times included: EX-HN3 and SP6 (see Figures 2, 3).

### Evaluation of methodological quality in selected records

Among the 23 included studies, six were rated as “low risk,” while 11 raised “some concerns” regarding the overall risk of bias. Six studies were judged to be at “high risk” of bias.

**Figure 4:**
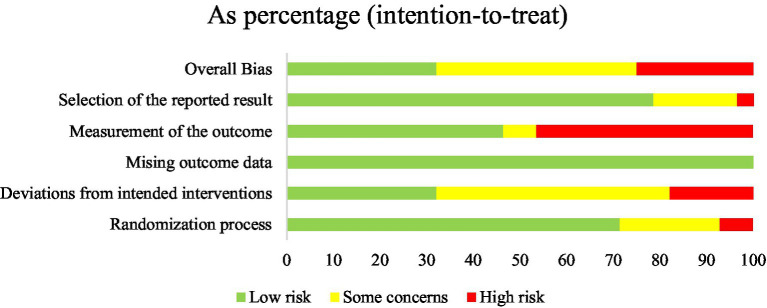
Risk of bias summary for studies.

**Figure 5:**
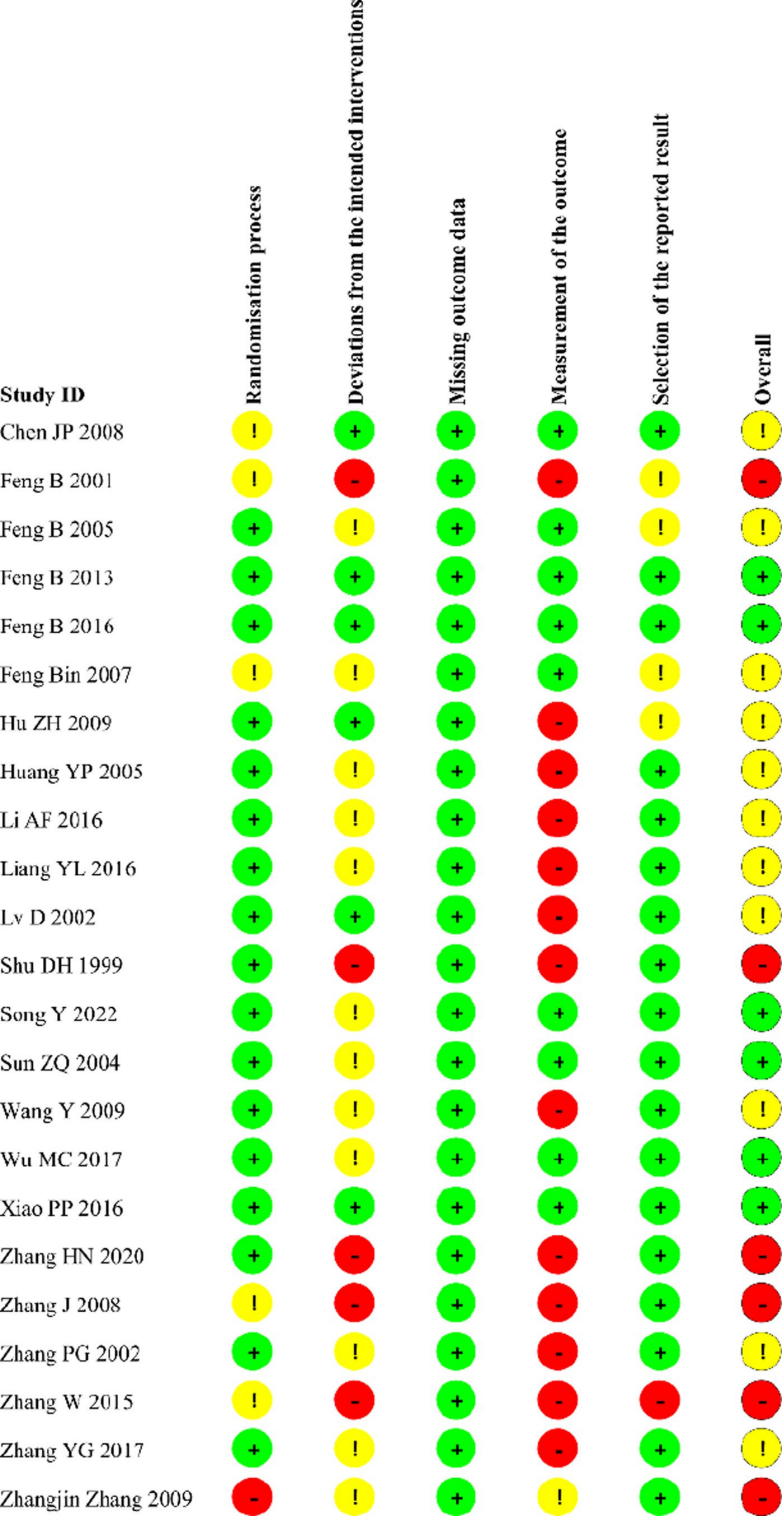
Risk of bias for each study.

Specifically, in the domain of the randomization process, 17 studies were at low risk, five raised some concerns, and one was at high risk. Regarding deviations from intended interventions, six studies were at low risk, 12 raised some concerns, and five were at high risk. All studies were at low risk for missing outcome data. In the measurement of outcomes, nine studies were at low risk, one raised some concerns, and 13 were at high risk. For the selection of the reported result, concerns were raised for four studies, and one study was at high risk (see Figures 4, 5).

### Effects of AS on Y-BOCS

**Figure 6:**
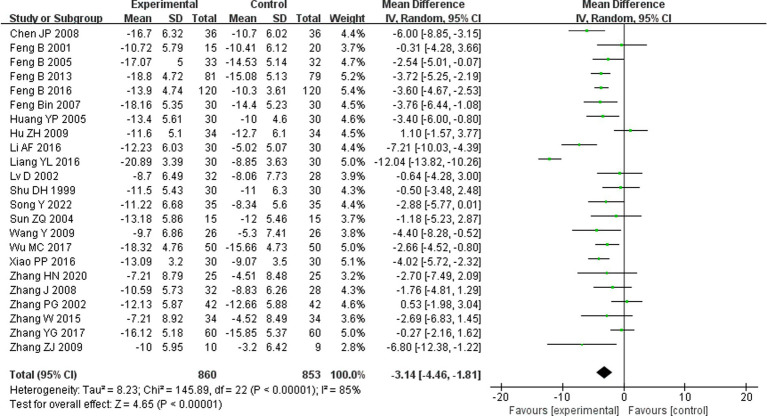
Y-BOCS.

**Figure 7:**
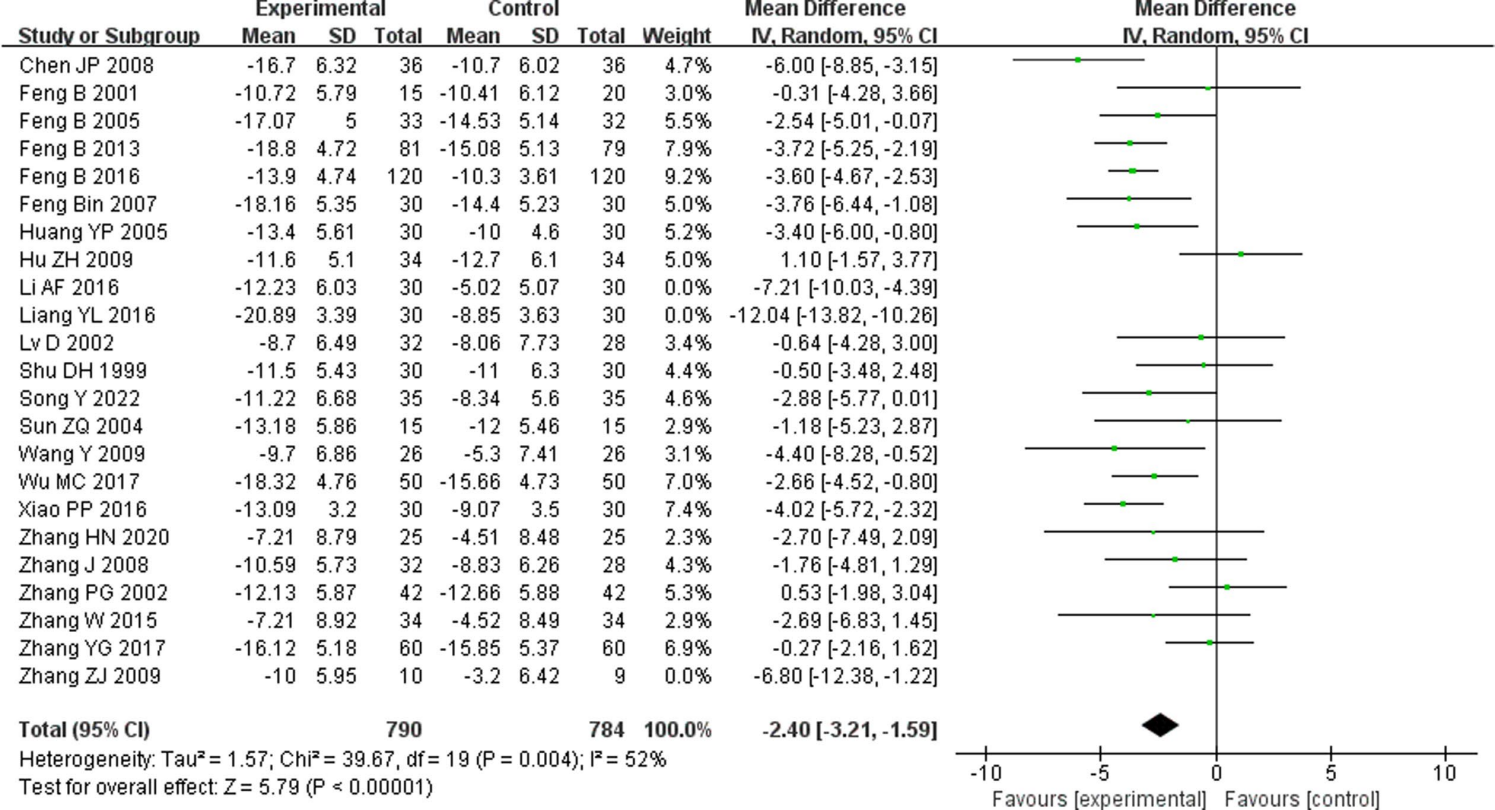
Sensitivity analysis of Y-BOCS.

Figure 6 demonstrates the significant effect of AS in reducing scores on the Yale-Brown Obsessive Compulsive Scale (Y-BOCS). Compared to control conditions—which included pharmacotherapy alone (*k* = 4), sham stimulation (*k* = 2), waitlist (*k* = 1), CBT-containing regimens (*k* = 3), and various combinations thereof (*k* = 13)—AS was associated with a statistically significant reduction in Y-BOCS scores (MD = −3.14, 95% CI [−4.46, −1.81], *p* < 0.00001). This pooled estimate should be interpreted with caution because the control conditions varied substantially across studies. Besides, a random-effects model was applied for the meta-analysis. A sensitivity analysis was performed by excluding the three studies [Zhang et al. (21), Li et al. (28), and Liang (29)] whose individual effect estimates deviated most from the pooled mean. After their removal, heterogeneity decreased from *I*^2^ = 85% to *I*^2^ = 52%, and the overall effect direction and significance were preserved (MD = −2.40, 95% CI [−3.21, −1.59], *p* < 0.00001; Figure 7). This suggests that the primary finding was not driven by a small set of outlying studies.

### Anxiety

**Figure 8:**
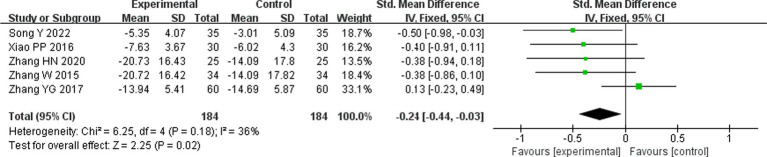
Anxiety.

The AS was associated with a small but statistically significant reduction in anxiety symptoms relative to control conditions (MD = −0.24, 95% CI [−0.44, −0.03], *p* = 0.02; Figure 8), with moderate heterogeneity (*I*^2^ = 59%, *p* = 0.18). The controls in this analysis comprised drug treatment alone and sham stimulation. As the effect size of a 0.24-point reduction is extremely small, the clinical significance of this reduction remains uncertain.

### BPRS

**Figure 9:**
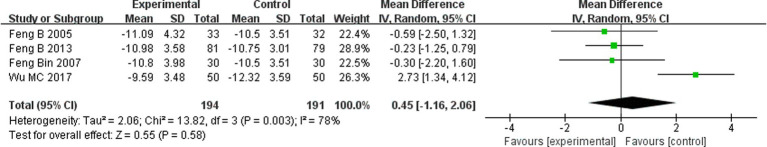
BPRS.

**Figure 10:**
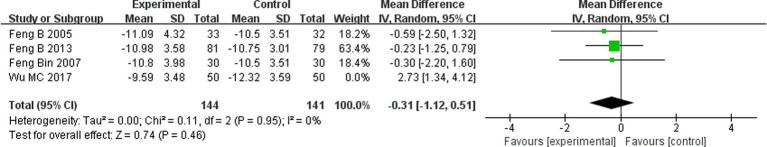
Sensitivity analysis of BPRS.

Four studies reported BPRS outcomes. AS was not associated with a significant reduction compared to control conditions (MD = −0.45, 95% CI [−1.16, 2.06]; *p* = 0.58; Figure 9), with substantial heterogeneity (*I*^2^ = 78%). A sensitivity analysis excluding the study whose effect estimate deviated most from the pooled mean resolved the heterogeneity (*I*^2^ = 0%) but did not change the null finding (Figure 10).

### CGI

**Figure 11:**
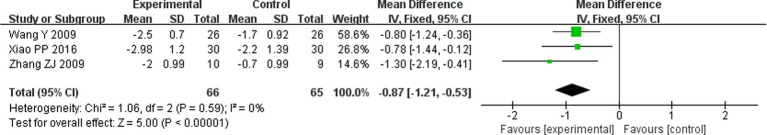
CGI.

Three studies reported CGI outcomes. AS was associated with a reduction compared to control conditions (MD = −0.87, 95% CI [−1.21, −0.53]; *p* < 0.001; Figure 11), with negligible heterogeneity (*I*^2^ = 0%). Given the small number of studies and the diverse control conditions (pharmacotherapy, waitlist, and sham stimulation), this estimate should be interpreted cautiously despite the statistical significance.

### Safety

**Figure 12:**
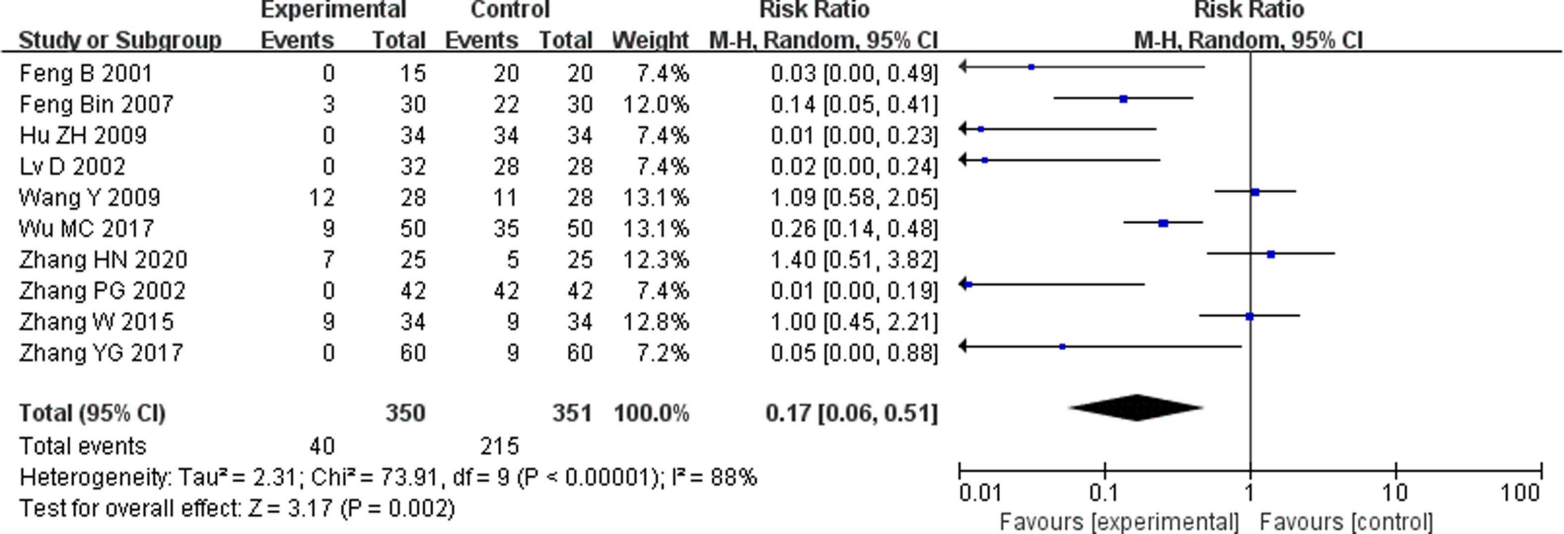
Safety.

Regarding safety, the AS group had fewer reported adverse events than medication controls (RR = 0.17, 95% CI [0.06, 0.51], *p* < 0.0001); however, the estimate varied by comparator drug, all safety data were derived from pharmacotherapy-controlled comparisons, and reporting was heterogeneous. A definitive safety conclusion is not warranted (Figure 12).

### Subgroup analysis for Y-BOCS

Subgroup analyses of the Y-BOCS were conducted based on the following three factors to explore potential sources of heterogeneity: intervention protocol, inclusion of PC6 in the acupoint protocol, and treatment frequency (Table 2).

**Table 2:** Subgroup analysis of Y-BOCS.

Subgroup type	Literature comparison group (n)	MD (95%CI)	*p*	Heterogeneity test	
				*I^2^*	*p*-value
Type of control					
AS vs. medicine	4	−1.75 (−4.61, 1.11)	0.23	63%	0.04
AS + CBT + medicine vs. other therapies	3	−3.50 (−4.34, −2.66)	<0.00001	0	0.51
AS + medicine vs. medicine	15	−2.97 (−5.19, −0.76)	0.009	91%	<0.00001
Whether it contains PC6					
Include PC6	17	−3.27 (−4.85, −1.69)	<0.0001	87%	<0.00001
Exclude PC6	6	−2.66 (−4.90, −0.42)	0.02	69%	0.007
Treatment frequency					
0–20 times	3	−3.11 (−6.11, −0.12)	0.04	45%	0.16
20–40 times	5	−3.86 (−8.47, 0.75)	0.10	96%	<0.00001
40–60 times	13	−2.63 (−3.89, −1.36)	<0.0001	64%	0.0008
>60 times	2	−3.66 (−4.68, −2.63)	<0.00001	0	0.7

### Publication bias

**Figure 13:**
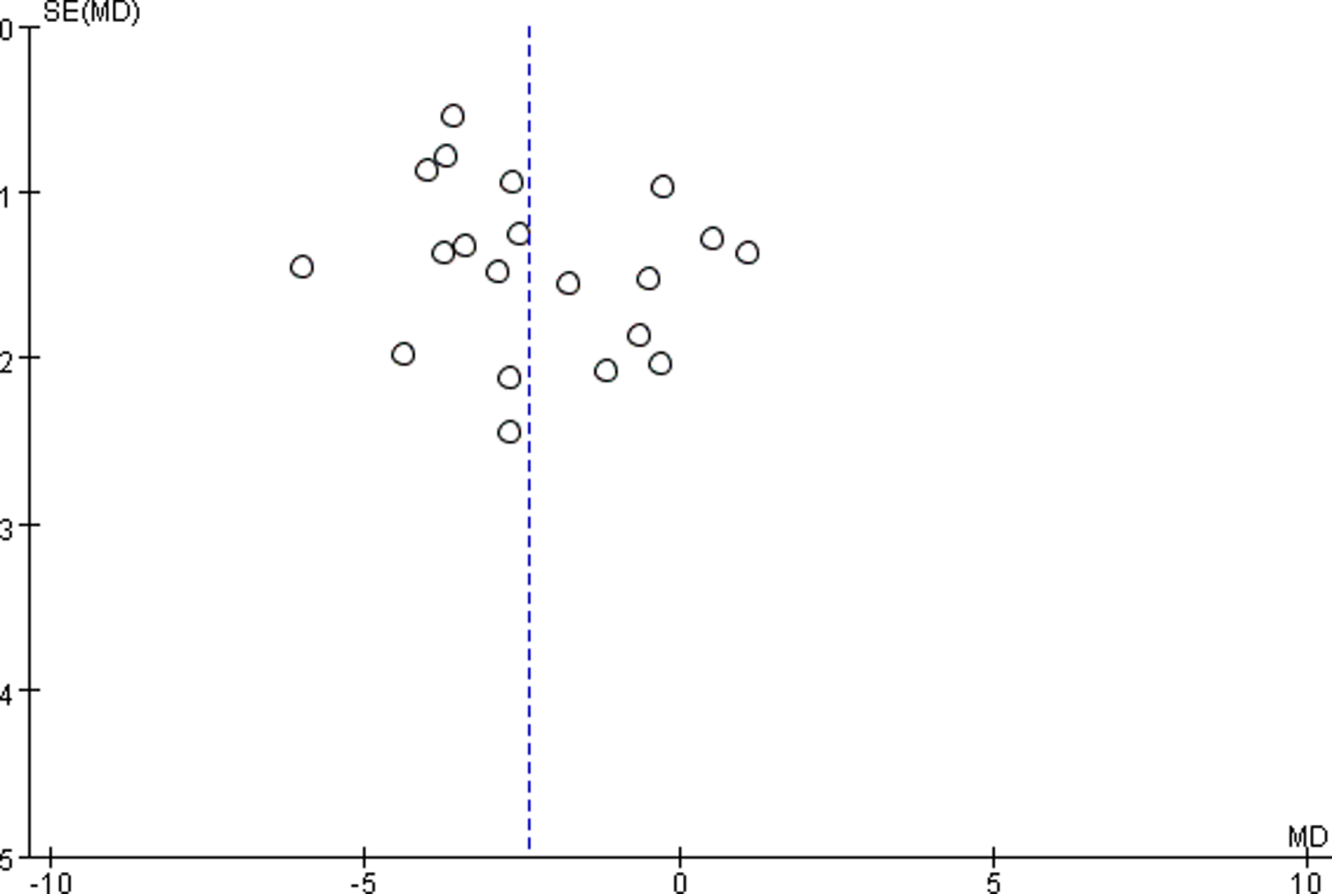
Publication bias for Y-BOCS.

The meta-analysis of Y-BOCS scores included more than 10 studies. Publication bias was assessed using a funnel plot. The funnel plot exhibited asymmetry, indicating a potential risk of publication bias (Figure 13).

## Discussion

The OCD has a complex, strongly heritable etiology involving genetic, neural, and environmental factors (42). Its core pathology is rooted in the cortico-striato-thalamo-cortical (CSTC) circuit. Here, an imbalance between the direct pathway (promoting actions) and the indirect pathway (inhibiting them) drives intrusive thoughts and compulsions (43, 44). Furthermore, dysregulated glutamate and GABA signaling within this network significantly contributes to the pathophysiology (45).

From a TCM perspective, AS is thought to regulate the balance of qi and blood along meridians (46). PC6, the luo-connecting point of the pericardium meridian, also communicates with the San Jiao meridian, which governs disorders arising from qi; needling PC6 is held to harmonize qi and blood, nourish the heart, and calm the shen (spirit) (25). GV20 (Baihui) is similarly used to tranquilize the mind and harmonize heart-brain function (32). The biological plausibility of AS for OCD, however, remains incompletely established. In preclinical and depression-related research, TEAS has been reported to modulate serotonergic and neuropeptidergic systems associated with the pathophysiology of OCD (47, 48). However, these findings are not specific to OCD and do not constitute direct mechanistic evidence. To date, no published study has examined the neurobiological pathways through which AS may affect obsessive-compulsive symptoms. This absence of OCD-specific mechanistic data represents an important gap: without a biologically plausible framework, statistically significant pooled effect estimates risk being misinterpreted as clinically meaningful. Besides, the included studies encompassed electroacupuncture, transcutaneous electrical stimulation, acupressure, and acupuncture, which differ in stimulation parameters and may not be clinically interchangeable; this heterogeneity is reflected in the primary analysis.

This systematic review and meta-analysis, comprising 23 RCTs (*n* = 1,713), evaluated AS as an adjunctive treatment for OCD. AS showed statistically significant associations with improvements in Y-BOCS, anxiety, and CGI scores. It was associated with greater improvements on these measures relative to control conditions, though effect sizes were modest and the primary outcome was accompanied by substantial heterogeneity. Furthermore, the clinical significance of the observed 3.14-point reduction is uncertain. On the Y-BOCS, which ranges from 0 to 40, a shift of this magnitude may represent only a modest change in symptom burden for many patients, particularly given the wide confidence interval. Moreover, the estimate was derived under substantial heterogeneity, meaning the true effect likely varies across patient subgroups and treatment protocols. These considerations reinforce the preliminary nature of the current evidence.

Sensitivity analyses for outcomes with high heterogeneity (*I*^2^ > 75%) could not pinpoint their source, which may stem from diverse AS protocols. Subgroup analyses were conducted. AS alone was not superior to pharmacotherapy alone (*p* > 0.05), but combining AS with pharmacotherapy yielded significantly greater benefits. The combination of AS with both pharmacotherapy and CBT yielded the largest Y-BOCS reduction among subgroups examined, which reduced Y-BOCS by a mean of 3.5 points more than the control (*p* < 0.05). Furthermore, studies that included PC6 showed a larger reduction (MD –3.27) than those that did not (MD –2.66), though the confidence intervals overlapped. In the exploratory subgroup analysis by treatment frequency, effect sizes varied across subgroups without a clear monotonic trend. The 20–40 session subgroup showed a reduction of MD –3.86 but did not reach statistical significance (*p* = 0.10) and was accompanied by extremely high heterogeneity (*I*^2^ = 96%). The >60 session subgroup showed a reduction of MD –3.66 (*p* < 0.05), though this is based on only two studies. These patterns are hypothesis-generating and should not be interpreted as evidence of a dose–response relationship.

Regarding acupoint selection, PC6 was the most frequently employed site (17 times), followed by GV20 (11 times).

All outcomes in this review were based on semi-structured scales. The Y-BOCS is a clinician-administered, semi-structured instrument. It comprises a symptom checklist and a severity scale to assess the intensity of obsessive-compulsive symptoms. For anxiety, we utilized the HAMA, a clinician-rated tool, and the SAS. Data from both scales were integrated to evaluate anxiety symptoms in OCD patients. The Clinical Global Impression (CGI) scale served as a global clinician-rated measure. It provides a rapid assessment of illness severity, therapeutic efficacy, and treatment-emergent adverse events.

Some research consistently supports SSRIs and CBT as first-line interventions for OCD (49, 50). The safety of other pharmacological agents—such as ketamine, memantine, lamotrigine, N-acetylcysteine, and ondansetron—remains unproven (51). This is true for their use either as monotherapy or in combination with SSRIs/CBT, though they offer alternative options for treatment-resistant patients (49, 52). Studies have explored neuromodulation techniques as adjunctive treatments for refractory OCD. These include deep brain stimulation (DBS), transcranial magnetic stimulation (TMS), transcranial direct current stimulation (tDCS), electroconvulsive therapy, and vagus nerve stimulation (53). Evidence suggests efficacy for patients unresponsive to pharmacotherapy and/or behavioral therapy. However, reported outcomes are often inconsistent, and standardized methodological protocols have not yet been established (53, 54).

### Limitation

However, this review has several limitations. First, the included trials are limited in number (*n* = 23) and accompanied by significant unexplained heterogeneity, which constrains the precision and generalizability of the pooled estimates. Second, the methodological quality of the primary studies was variable—only six were rated as low risk of bias—and potential publication bias further limits confidence in the findings. Third, partial sample overlap within the Feng study cluster cannot be definitively excluded; a cross-comparison is provided in Supplementary Table S1. Fourth, all 23 trials were conducted in China, and the generalizability of the findings to other populations and healthcare settings is uncertain. Fifth, no published study has directly examined the neurobiological mechanism of AS in OCD populations; the biological plausibility of AS for this indication therefore remains speculative. Sixth, all safety data were derived from comparisons against pharmacotherapy controls; the safety advantage varied by comparator drug, and no safety data from sham-controlled or non-pharmacological comparisons were available. The subgroup analyses were post-hoc and exploratory and should be regarded as hypothesis-generating. Future research should prioritize high-quality, prospectively registered, multicenter RCTs with standardized AS protocols, systematic adverse-event monitoring, and OCD-specific mechanistic investigations.

## Conclusion

This review of 23 RCTs provides preliminary evidence that AS, whether used alone or combined with pharmacotherapy or CBT, is associated with statistically significant but modest reductions in Y-BOCS scores. The clinical significance of this reduction is uncertain, and the evidence is constrained by substantial heterogeneity, variable methodological quality, potential publication bias, possible sample overlap within the Feng study cluster, and the concentration of all trials in a single country. The available safety data preclude a definitive safety conclusion. These findings do not yet support a general recommendation for AS as an established treatment for OCD; rather, they indicate a signal of potential benefit that requires confirmation in rigorously designed multicenter RCTs.

## Data Availability

The original contributions presented in the study are included in the article/Supplementary material, further inquiries can be directed to the corresponding author.
