# Supplementary material for: The effect of acupoint stimulation as an adjuvant therapy for obsessive-compulsive disorder: a systematic review and meta-analysis
Source: Front Neurol. 2026 Jul 23;17:1875285. doi: 10.3389/fneur.2026.1875285 (PMC13441982; doi:10.3389/fneur.2026.1875285)
Supplement: Supplementary file 1 [file Table_1.DOCX]

**Wanfang:**

期刊、学位、会议	主题:(针刺 or 针灸 or 推拿 or 艾灸 or 穴位按压 or 电针 or 经皮穴位电刺激 or 穴位贴敷 or 耳穴 or 穴位刺激) and 主题:(强迫症 or 强迫行为 or 强迫思维 or 强迫性神经症 or 强迫症状)

**CBM：**

序号	检索表达式	命中文献数	检索时间

1)	((((("针灸康复"[不加权:扩展]) OR "针灸疗法"[不加权:扩展] OR "温针疗法"[不加权:扩展] OR "逆针灸"[不加权:扩展] OR "微波针刺疗法"[不加权:扩展] OR "太乙针灸疗法"[不加权:扩展] OR "针灸处方"[不加权:扩展] OR "针灸疗法"[不加权:扩展] OR "针灸效应"[不加权:扩展]) OR "推拿疗法"[不加权:扩展]) OR "针刺"[不加权:扩展] OR "针刺, 耳"[不加权:扩展] OR "微波针刺疗法"[不加权:扩展] OR "针刺泻法"[不加权:扩展] OR "针刺补法"[不加权:扩展]) OR "穴位, 头针刺激区"[不加权:扩展] OR "穴位, 头针刺激区"[不加权:扩展]) OR "点穴疗法"[不加权:扩展] OR "穴位按压"[不加权:扩展]	249080	2026-02-01 00:57:47.0

2)	("强迫性障碍"[不加权:扩展] OR "囤积症"[不加权:扩展]) OR "强迫行为"[不加权:扩展]

3)	"经皮穴位电刺激"[常用字段:智能] OR "穴位刺激"[常用字段:智能]

4)	("经皮穴位电刺激"[常用字段:智能] OR "穴位刺激"[常用字段:智能]) OR (((((("针灸康复"[不加权:扩展]) OR "针灸疗法"[不加权:扩展] OR "温针疗法"[不加权:扩展] OR "逆针灸"[不加权:扩展] OR "微波针刺疗法"[不加权:扩展] OR "太乙针灸疗法"[不加权:扩展] OR "针灸处方"[不加权:扩展] OR "针灸疗法"[不加权:扩展] OR "针灸效应"[不加权:扩展]) OR "推拿疗法"[不加权:扩展]) OR "针刺"[不加权:扩展] OR "针刺, 耳"[不加权:扩展] OR "微波针刺疗法"[不加权:扩展] OR "针刺泻法"[不加权:扩展] OR "针刺补法"[不加权:扩展]) OR "穴位, 头针刺激区"[不加权:扩展] OR "穴位, 头针刺激区"[不加权:扩展]) OR "点穴疗法"[不加权:扩展] OR "穴位按压"[不加权:扩展])

5)	(("经皮穴位电刺激"[常用字段:智能] OR "穴位刺激"[常用字段:智能]) OR (((((("针灸康复"[不加权:扩展]) OR "针灸疗法"[不加权:扩展] OR "温针疗法"[不加权:扩展] OR "逆针灸"[不加权:扩展] OR "微波针刺疗法"[不加权:扩展] OR "太乙针灸疗法"[不加权:扩展] OR "针灸处方"[不加权:扩展] OR "针灸疗法"[不加权:扩展] OR "针灸效应"[不加权:扩展]) OR "推拿疗法"[不加权:扩展]) OR "针刺"[不加权:扩展] OR "针刺, 耳"[不加权:扩展] OR "微波针刺疗法"[不加权:扩展] OR "针刺泻法"[不加权:扩展] OR "针刺补法"[不加权:扩展]) OR "穴位, 头针刺激区"[不加权:扩展] OR "穴位, 头针刺激区"[不加权:扩展]) OR "点穴疗法"[不加权:扩展] OR "穴位按压"[不加权:扩展])) AND (("强迫性障碍"[不加权:扩展] OR "囤积症"[不加权:扩展]) OR "强迫行为"[不加权:扩展])

**CNKI：**

（主题：强迫症 + 强迫行为 + 强迫思维 + 强迫性神经症 + 强迫症状）AND（主题：针刺 + 针灸 + 推拿 + 艾灸 + 穴位按压 + 电针 + 经皮穴位电刺激 + 穴位贴敷 + 耳穴 + 穴位刺激）	资源范围：学术期刊; 仅看有全文,中英文扩展; 时间范围：更新时间：不限; 来源类别：全部期刊;

**Cochrane:**

Search Name:

Date Run:	01/02/2026 15:20:29

Comment:

ID	Search	Hits

#1	MeSH descriptor: [Acupuncture Points] explode all trees

#2	MeSH descriptor: [Acupuncture] explode all trees

#3	MeSH descriptor: [Acupuncture Therapy] explode all trees

#4	MeSH descriptor: [Acupuncture, Ear] explode all trees

#5	MeSH descriptor: [Meridians] explode all trees

#6	MeSH descriptor: [Acupressure] explode all trees

#7	MeSH descriptor: [Electroacupuncture] explode all trees

#8	MeSH descriptor: [Transcutaneous Electric Nerve Stimulation] explode all trees

#9	(Transcutaneous Nerve Stimulation):ab,ti,kw OR (acupoint stimulation):ab,ti,kw

#10	#1 OR #2 OR #3 OR #4 OR #5 OR #6 OR #7 OR #8 OR #9

#11	MeSH descriptor: [Obsessive-Compulsive Disorder] explode all trees

#12	(Disorder, Obsessive-Compulsive):ab,ti,kw OR (Anankastic Personality):ab,ti,kw OR (Obsessive-Compulsive Neuroses)

#13	#11 OR #12

#14	#10 AND #13

**Embase:**

**Medline:**

(((Transcutaneous Nerve Stimulation) OR (acupoint stimulation)) OR (((((("Acupuncture Points"[Mesh]) OR ( "Acupuncture"[Mesh] OR "Acupuncture Therapy"[Mesh] OR "Acupuncture, Ear"[Mesh] )) OR "Meridians"[Mesh]) OR "Acupressure"[Mesh]) OR "Electroacupuncture"[Mesh]) OR "Transcutaneous Electric Nerve Stimulation"[Mesh])) AND ((((Disorder, Obsessive-Compulsive) OR (Anankastic Personality)) OR (Obsessive-Compulsive Neuroses)) OR ("Obsessive-Compulsive Disorder"[Mesh]))

**Web of science:**

3/3

3

#1 AND #2

2

Disorder, Obsessive-Compulsive (Topic) or obsessive compulsive disorder (Topic) or compulsion (Topic) or obsession (Topic) or refractory obsessive compulsive disorder (Topic) or Anankastic Personality (Topic) or Obsessive-Compulsive Disorder (Topic) or Obsessive-Compulsive Neuroses (Topic)

1

Acupoint stimulation (Topic) or acupuncture (Topic) or tuina (Topic) or Transcutaneous Nerve Stimulation (Topic) or Acupuncture Points (Topic) or Acupuncture, Ear (Topic) or Acupressure (Topic) or Meridians (Topic) or Electroacupuncture (Topic) or Transcutaneous Electric Nerve Stimulation (Topic)
