# Supplementary material for: The effect of acupoint stimulation as an adjuvant therapy for obsessive-compulsive disorder: a systematic review and meta-analysis
Source: Front Neurol. 2026 Jul 23;17:1875285. doi: 10.3389/fneur.2026.1875285 (PMC13441982; doi:10.3389/fneur.2026.1875285)
Supplement: Supplementary file 2 [file Table_2.DOCX]

**Post-hoc sensitivity analysis**

Even if we adopt the most conservative assumption—that there may be complete overlap between all the studies cited by Bin Feng’s team, and we retain only the largest one—the core conclusions remain essentially unchanged.
