# Supplementary material for: The effect of acupoint stimulation as an adjuvant therapy for obsessive-compulsive disorder: a systematic review and meta-analysis
Source: Front Neurol. 2026 Jul 23;17:1875285. doi: 10.3389/fneur.2026.1875285 (PMC13441982; doi:10.3389/fneur.2026.1875285)
Supplement: Supplementary file 3 [file Table_3.DOCX]

**Chinese Article Original Language Titles**

16. 董峰,吕艳斐.中药配合电针治疗强迫症验案一则[J].实用中医药杂志,2018,34(8):1001-1002.

17. 樊志奇,周嘉欣,徐书君,等.符文彬教授针灸治疗强迫症经验总结[J].中国老年学杂志,2013,33(9):2230-2231.

18. 农秋葵,李思珏,熊令辉.临床护理路径在针灸治疗强迫症患者护理中的应用[J].齐鲁护理杂志,2017,23(13):17-19.

22. 陈建萍,叶锋,曾为群.电针合并氟西汀治疗精神分裂症伴强迫症状36例[J].中国中西医结合杂志,2008,(6):561-562.

23. 冯斌.穴位刺激调控法治疗强迫症的临床研究[J].浙江中西医结合杂志,2001,(10):19-20.

24. 冯斌,刘兰英,徐方忠,等.穴位刺激调控法治疗强迫症患者的临床观察[J].中国中西医结合杂志,2005,(9):801-803.

25. 冯斌,朱锐明,徐方忠,等.穴位刺激调控法联合小剂量氯米帕明片治疗强迫症81例随机双盲临床研究[J].中医杂志,2013,54(9):762-765.

26. 胡振宏,赵安全,康世东.针刺下脱敏疗法治疗焦虑症强迫症临床对照研究[J].中国医药导刊,2009,11(2):317-318.

27. 黄寅平,姚建军,季庆.穴位刺激调控法合并氟西汀治疗强迫症[J].中国康复,2005,(4):251-252.

28. 李爱凤,徐文军,张双春. 帕罗西汀联合针刺治疗难治性强迫症的对照研究[J]. 国际医药卫生导报,2016,22(16):2406-2408.

29. 梁银利. 针刺对强迫症患者反应抑制能力的影响[J]. 医药前沿,2016,6(27):298-299.

30. 吕多.针刺与氯丙咪嗪治疗强迫症疗效对比观察[J].中国针灸,2002,(8):16-17.

31. 舒德海,周桂芝,何华,等.电针为主治疗强迫症30例疗效观察[J].中国针灸,1999,(12):9-10+4.

32. 宋阳,王恩忠,文丹阳,等.疏肝调神针法联合三神穴长留针治疗强迫症的疗效观察[J].上海针灸杂志,2022,41(9):878-882.

33. 孙兆庆,祝英禄,李洪祥.电针及氯米帕明并用治疗强迫症(英文)[J].中国临床康复,2005,(8):218-219.

34. 汪艳,周振和,姚建军.穴位刺激调控法合并氟伏沙明治疗强迫症的疗效观察[J].精神医学杂志,2009,22(5):391-392.

35. 伍明超,丁亚文.穴位刺激联合小剂量氯米帕明治疗强迫症疗效观察[J].现代中西医结合杂志,2017,26(21):2360-2362.

36. 肖攀攀,曹叶群,农秋葵,等.针灸辅助帕罗西汀治疗强迫症的临床疗效观察[J].中国临床研究,2016,29(8):1123-1125.

37. 张赫男. 清心除烦针灸疗法配合氟伏沙明对强迫症的治疗作用及安全性分析[J]. 中国保健营养,2020,30(32):328-329.

38. 张静,刘爱红.认知疗法结合电针治疗强迫症临床疗效[J].中国误诊学杂志,2008,(30):7362-7363.

39. 张平根,钟旗,余艳娥,等.电针与氯米帕明治疗强迫症的对照观察[J].中国中西医结合杂志,2002,(3):215-216.

40. 张旺,赵安全,马建华,等.清心除烦针灸疗法配合氟伏沙明治疗强迫症疗效分析[J].河北中医药学报,2015,30(3):60-62.

41. 张永国,郭晓娟.丁螺环酮与醒脑开窍针刺法治疗强迫症的疗效对比[J].西部中医药,2017,30(2):98-99.
