# Supplementary material for: The effect of acupoint stimulation as an adjuvant therapy for obsessive-compulsive disorder: a systematic review and meta-analysis
Source: Front Neurol. 2026 Jul 23;17:1875285. doi: 10.3389/fneur.2026.1875285 (PMC13441982; doi:10.3389/fneur.2026.1875285)
Supplement: Supplementary file 4 [file Table_4.DOCX]

**1. Wanfang:**

期刊、学位、会议	主题:(针刺 or 针灸 or 推拿 or 艾灸 or 穴位按压 or 电针 or 经皮穴位电刺激 or 穴位贴敷 or 耳穴 or 穴位刺激) and 主题:(强迫症 or 强迫行为 or 强迫思维 or 强迫性神经症 or 强迫症状)

Journals, Theses, Conferences Subject: (acupuncture or moxibustion or Tuina or acupressure or electroacupuncture or transcutaneous electrical nerve stimulation or acupoint plasters or auricular acupuncture or acupoint stimulation) AND Subject: (obsessive-compulsive disorder or compulsive behaviour or obsessive thoughts or obsessive-compulsive neurosis or obsessive-compulsive symptoms)

**2. CBM：**

序号	检索表达式	命中文献数	检索时间

1)	((((("针灸康复"[不加权:扩展]) OR "针灸疗法"[不加权:扩展] OR "温针疗法"[不加权:扩展] OR "逆针灸"[不加权:扩展] OR "微波针刺疗法"[不加权:扩展] OR "太乙针灸疗法"[不加权:扩展] OR "针灸处方"[不加权:扩展] OR "针灸疗法"[不加权:扩展] OR "针灸效应"[不加权:扩展]) OR "推拿疗法"[不加权:扩展]) OR "针刺"[不加权:扩展] OR "针刺, 耳"[不加权:扩展] OR "微波针刺疗法"[不加权:扩展] OR "针刺泻法"[不加权:扩展] OR "针刺补法"[不加权:扩展]) OR "穴位, 头针刺激区"[不加权:扩展] OR "穴位, 头针刺激区"[不加权:扩展]) OR "点穴疗法"[不加权:扩展] OR "穴位按压"[不加权:扩展]	249080	2026-02-01 00:57:47.0

2)	("强迫性障碍"[不加权:扩展] OR "囤积症"[不加权:扩展]) OR "强迫行为"[不加权:扩展]

3)	"经皮穴位电刺激"[常用字段:智能] OR "穴位刺激"[常用字段:智能]

4)	("经皮穴位电刺激"[常用字段:智能] OR "穴位刺激"[常用字段:智能]) OR (((((("针灸康复"[不加权:扩展]) OR "针灸疗法"[不加权:扩展] OR "温针疗法"[不加权:扩展] OR "逆针灸"[不加权:扩展] OR "微波针刺疗法"[不加权:扩展] OR "太乙针灸疗法"[不加权:扩展] OR "针灸处方"[不加权:扩展] OR "针灸疗法"[不加权:扩展] OR "针灸效应"[不加权:扩展]) OR "推拿疗法"[不加权:扩展]) OR "针刺"[不加权:扩展] OR "针刺, 耳"[不加权:扩展] OR "微波针刺疗法"[不加权:扩展] OR "针刺泻法"[不加权:扩展] OR "针刺补法"[不加权:扩展]) OR "穴位, 头针刺激区"[不加权:扩展] OR "穴位, 头针刺激区"[不加权:扩展]) OR "点穴疗法"[不加权:扩展] OR "穴位按压"[不加权:扩展])

5)	(("经皮穴位电刺激"[常用字段:智能] OR "穴位刺激"[常用字段:智能]) OR (((((("针灸康复"[不加权:扩展]) OR "针灸疗法"[不加权:扩展] OR "温针疗法"[不加权:扩展] OR "逆针灸"[不加权:扩展] OR "微波针刺疗法"[不加权:扩展] OR "太乙针灸疗法"[不加权:扩展] OR "针灸处方"[不加权:扩展] OR "针灸疗法"[不加权:扩展] OR "针灸效应"[不加权:扩展]) OR "推拿疗法"[不加权:扩展]) OR "针刺"[不加权:扩展] OR "针刺, 耳"[不加权:扩展] OR "微波针刺疗法"[不加权:扩展] OR "针刺泻法"[不加权:扩展] OR "针刺补法"[不加权:扩展]) OR "穴位, 头针刺激区"[不加权:扩展] OR "穴位, 头针刺激区"[不加权:扩展]) OR "点穴疗法"[不加权:扩展] OR "穴位按压"[不加权:扩展])) AND (("强迫性障碍"[不加权:扩展] OR "囤积症"[不加权:扩展]) OR "强迫行为"[不加权:扩展])

No. Search Term	Number of Relevant Documents	Search Date

1)	(((((‘Acupuncture Rehabilitation’[unweighted: expanded]) OR ‘Acupuncture Therapy’[unweighted: expanded] OR ‘Warm Needle Therapy’[unweighted: expanded] OR ‘Reverse Acupuncture’[unweighted: expanded] OR ‘Microwave Acupuncture Therapy’ [unweighted: expanded] OR “Taiyi Acupuncture Therapy”[unweighted: expanded] OR “Acupuncture Prescriptions”[unweighted: expanded] OR “Acupuncture Therapy”[unweighted: expanded] OR “Acupuncture Effects” [unweighted: expanded]) OR ‘Tuina therapy’[unweighted: expanded]) OR “acupuncture”[unweighted: expanded] OR ‘acupuncture, auricular’[unweighted: expanded] OR ‘microwave acupuncture therapy’[unweighted: expanded] OR ‘acupuncture, sedation method’ [unweighted: expanded] OR ‘acupuncture tonification method’[unweighted: expanded]) OR ‘acupoints, scalp acupuncture stimulation zones’[unweighted: expanded] OR ‘acupoints, scalp acupuncture stimulation zones’[unweighted: expanded]) OR ‘acupoint therapy’[unweighted: expanded] OR ‘acupoint pressure’[unweighted: expanded]	249080	1 February 2026 00:57:47.0

2)	(‘Obsessive-compulsive disorder’ [Unweighted: Expanded] OR ‘Hoarding disorder’ [Unweighted: Expanded]) OR ‘Compulsive behaviour’ [Unweighted: Expanded]

3)	‘Transcutaneous electrical acupoint stimulation’[Common field: Smart] OR ‘Acupoint stimulation’[Common field: Smart]

4)	(‘Transcutaneous electrical acupoint stimulation’[Common field: Smart] OR ‘Acupoint stimulation’[Common field: Smart]) OR ((((((‘Acupuncture rehabilitation’[Unweighted: Expanded]) OR ‘Acupuncture therapy’ [Unweighted: Expanded] OR ‘Warm-needle therapy’[Unweighted: Expanded] OR ‘Reverse acupuncture’[Unweighted: Expanded] OR ‘Microwave acupuncture therapy’[Unweighted: Expanded] OR ‘Taiyi acupuncture therapy’[Unweighted: Expanded] OR ‘Acupuncture prescription’[Unweighted: Expanded] OR ‘Acupuncture therapy’ [unweighted: expanded] OR ‘acupuncture effects’[unweighted: expanded]) OR ‘Tuina therapy’[unweighted: expanded]) OR “acupuncture”[unweighted: expanded] OR ‘acupuncture, auricular’[unweighted: expanded] OR ‘microwave acupuncture therapy’[unweighted: expanded] OR ‘acupuncture dispersion method’ [Unweighted: Expanded] OR ‘acupuncture tonification method’[Unweighted: Expanded]) OR ‘acupoints, scalp acupuncture stimulation zones’[Unweighted: Expanded] OR ‘acupoints, scalp acupuncture stimulation zones’[Unweighted: Expanded]) OR ‘acupoint therapy’[Unweighted: Expanded] OR ‘acupoint pressure’[Unweighted: Expanded])

5)	((‘Transcutaneous electrical acupoint stimulation’[Common field: Smart] OR ‘Acupoint stimulation’[Common field: Smart]) OR ((((((" Acupuncture rehabilitation‘[unweighted: expanded]) OR ’Acupuncture therapy‘[unweighted: expanded] OR ’Warm needle therapy‘[unweighted: expanded] OR ’Reverse acupuncture‘[unweighted: expanded] OR ’Microwave acupuncture therapy‘[unweighted: expanded] OR ’Taiyi acupuncture therapy‘[unweighted: expanded] OR ’Acupuncture prescription" [unweighted: expanded] OR ‘acupuncture therapy’[unweighted: expanded] OR ‘acupuncture effects’[unweighted: expanded]) OR ‘Tuina therapy’[unweighted: expanded]) OR “acupuncture”[unweighted: expanded] OR ‘acupuncture, auricular’[unweighted: expanded] OR ‘microwave acupuncture therapy’ [Unweighted: Expanded] OR ‘Acupuncture Discharge Method’[Unweighted: Expanded] OR ‘Acupuncture Tonification Method’[Unweighted: Expanded]) OR ‘Acupoints, Scalp Acupuncture Stimulation Zones’[Unweighted: Expanded] OR ‘Acupoints, Scalp Acupuncture Stimulation Zones’ [unweighted: expanded]) OR ‘acupressure therapy’[unweighted: expanded] OR ‘acupressure point stimulation’[unweighted: expanded])) AND ((‘obsessive-compulsive disorder’[unweighted: expanded] OR ‘hoarding disorder’[unweighted: expanded]) OR ‘compulsive behaviour’[unweighted: expanded])

**3. CNKI：**

（主题：强迫症 + 强迫行为 + 强迫思维 + 强迫性神经症 + 强迫症状）AND（主题：针刺 + 针灸 + 推拿 + 艾灸 + 穴位按压 + 电针 + 经皮穴位电刺激 + 穴位贴敷 + 耳穴 + 穴位刺激）	资源范围：学术期刊; 仅看有全文,中英文扩展; 时间范围：更新时间：不限; 来源类别：全部期刊;

(Subject: Obsessive-compulsive disorder + Compulsive behaviour + Obsessive thoughts + Obsessive-compulsive neurosis + Obsessive-compulsive symptoms) AND (Subject: Acupuncture + Acupressure + Tuina + Moxibustion + Acupoint pressure + Electroacupuncture + Transcutaneous electrical nerve stimulation (TENS) + Acupoint plaster therapy + Auricular acupuncture + Acupoint stimulation) Resource scope: Academic journals; Full-text only, Chinese and English; Time range: Update date: Any; Source type: All journals;

**4. Cochrane:**

Search Name:

Date Run:	01/02/2026 15:20:29

Comment:

ID	Search	Hits

#1	MeSH descriptor: [Acupuncture Points] explode all trees

#2	MeSH descriptor: [Acupuncture] explode all trees

#3	MeSH descriptor: [Acupuncture Therapy] explode all trees

#4	MeSH descriptor: [Acupuncture, Ear] explode all trees

#5	MeSH descriptor: [Meridians] explode all trees

#6	MeSH descriptor: [Acupressure] explode all trees

#7	MeSH descriptor: [Electroacupuncture] explode all trees

#8	MeSH descriptor: [Transcutaneous Electric Nerve Stimulation] explode all trees

#9	(Transcutaneous Nerve Stimulation):ab,ti,kw OR (acupoint stimulation):ab,ti,kw

#10	#1 OR #2 OR #3 OR #4 OR #5 OR #6 OR #7 OR #8 OR #9

#11	MeSH descriptor: [Obsessive-Compulsive Disorder] explode all trees

#12	(Disorder, Obsessive-Compulsive):ab,ti,kw OR (Anankastic Personality):ab,ti,kw OR (Obsessive-Compulsive Neuroses)

#13	#11 OR #12

#14	#10 AND #13

**5. Embase:**

**6. Medline:**

(((Transcutaneous Nerve Stimulation) OR (acupoint stimulation)) OR (((((("Acupuncture Points"[Mesh]) OR ( "Acupuncture"[Mesh] OR "Acupuncture Therapy"[Mesh] OR "Acupuncture, Ear"[Mesh] )) OR "Meridians"[Mesh]) OR "Acupressure"[Mesh]) OR "Electroacupuncture"[Mesh]) OR "Transcutaneous Electric Nerve Stimulation"[Mesh])) AND ((((Disorder, Obsessive-Compulsive) OR (Anankastic Personality)) OR (Obsessive-Compulsive Neuroses)) OR ("Obsessive-Compulsive Disorder"[Mesh]))

**7. Web of science:**

3/3

3

#1 AND #2

2

Disorder, Obsessive-Compulsive (Topic) or obsessive compulsive disorder (Topic) or compulsion (Topic) or obsession (Topic) or refractory obsessive compulsive disorder (Topic) or Anankastic Personality (Topic) or Obsessive-Compulsive Disorder (Topic) or Obsessive-Compulsive Neuroses (Topic)

1

Acupoint stimulation (Topic) or acupuncture (Topic) or tuina (Topic) or Transcutaneous Nerve Stimulation (Topic) or Acupuncture Points (Topic) or Acupuncture, Ear (Topic) or Acupressure (Topic) or Meridians (Topic) or Electroacupuncture (Topic) or Transcutaneous Electric Nerve Stimulation (Topic)
