# Supplementary material for: The effect of acupoint stimulation as an adjuvant therapy for obsessive-compulsive disorder: a systematic review and meta-analysis
Source: Front Neurol. 2026 Jul 23;17:1875285. doi: 10.3389/fneur.2026.1875285 (PMC13441982; doi:10.3389/fneur.2026.1875285)
Supplement: Supplementary file 5 [file Table_5.DOCX]

| Article | Feng B 2001 | Feng B 2005 | Feng B 2013 | Feng Bin 2016 | Feng Bin 2007 |
| --- | --- | --- | --- | --- | --- |
| Years | 2001 | 2005 | 2013 | 2016 | 2007 |
| Sample size | EA + Chlorimipramine (n=15)  Chlorimipramine (n=20) | EA + CBT (n=33)  Chlorimipramine (n=32) | EA + CBT + Chlorimipramine (n=81)  sham EA + Chlorimipramine (n=79) | TEAS + CBT + clomipramine (n=120)  Sham TEAS + CBT + clomipramine (n=120)  cognitive behavioral therapy (CBT)  transcutaneous electrical acupoint  stimulation (TEAS) | EA + Chlorimipramine (n=30)  Chlorimipramine (n=30) |
| Gender (male/ female) | T: 9/6  C: 12/8 | T:14/18  C:15/18 | T:34/47  C:46/33 | T: 52/68  C: 52/68 | T: 13/17  C: 13/17 |
| Disease duration | T: 5.5 years  C: 5.4 years | T: 65 months  C: 66 months | T: 66 months  C: 54 months | T: 1-10 years  C: 1-10 years | T: 65 months  C: 66 months |
| reference | 冯斌.穴位刺激调控法治疗强迫症的临床研究[J].浙江中西医结合杂志,2001,(10):19-20. | 冯斌,刘兰英,徐方忠,等.穴位刺激调控法治疗强迫症患者的临床观察[J].中国中西医结合杂志,2005,(9):801-803. | 冯斌,朱锐明,徐方忠,等.穴位刺激调控法联合小剂量氯米帕明片治疗强迫症81例随机双盲临床研究[J].中医杂志,2013,54(9):762-765. | Feng B, Zhang ZJ, Zhu RM, et al. Transcutaneous electrical acupoint stimulation as an adjunct therapy for obsessive-compulsive disorder: A randomized controlled study. Journal of psychiatric research. 2016;80:30-7. doi: 10.1016/j.jpsychires.2016.05.015 | Bin F, Lanying L, Fangzhong X, et al. Thirty cases of obsession treated by point-stimulation and with small dose of chlorimipramine. Journal of Traditional Chinese Medicine. 2007;27(1):3-6. PMID: 17393613 |

**Bin Feng study cluster**
